# Recent Advances in the Roles of HSFs and HSPs in Heat Stress Response in Woody Plants

**DOI:** 10.3389/fpls.2021.704905

**Published:** 2021-07-09

**Authors:** Fengxia Tian, Xiao-Li Hu, Tao Yao, Xiaohan Yang, Jin-Gui Chen, Meng-Zhu Lu, Jin Zhang

**Affiliations:** ^1^College of Life Science and Agricultural Engineering, Nanyang Normal University, Nanyang, China; ^2^State Key Laboratory of Subtropical Silviculture, College of Forestry and Biotechnology, Zhejiang A&F University, Hangzhou, China; ^3^Biosciences Division, Oak Ridge National Laboratory, Oak Ridge, TN, United States

**Keywords:** heat stress, woody plants, signaling network, molecular response, heat shock transcription factor, heat shock protein

## Abstract

A continuous increase in ambient temperature caused by global warming has been considered a worldwide threat. As sessile organisms, plants have evolved sophisticated heat shock response (HSR) to respond to elevated temperatures and other abiotic stresses, thereby minimizing damage and ensuring the protection of cellular homeostasis. In particular, for perennial trees, HSR is crucial for their long life cycle and development. HSR is a cell stress response that increases the number of chaperones including heat shock proteins (HSPs) to counter the negative effects on proteins caused by heat and other stresses. There are a large number of HSPs in plants, and their expression is directly regulated by a series of heat shock transcription factors (HSFs). Therefore, understanding the detailed molecular mechanisms of woody plants in response to extreme temperature is critical for exploring how woody species will be affected by climate changes. In this review article, we summarize the latest findings of the role of HSFs and HSPs in the HSR of woody species and discuss their regulatory networks and cross talk in HSR. In addition, strategies and programs for future research studies on the functions of HSFs and HSPs in the HSR of woody species are also proposed.

## Introduction

As one of the most harmful abiotic stresses, heat stress poses a threat to plant life by impacting plant metabolism directly (Rennenberg et al., 2006). In order to successfully survive the occurrence of a long heatwave, plants have evolved a series of adaptive mechanisms to cope with the rising ambient temperature (Bäurle, 2016). Among those mechanisms, heat shock response (HSR) is a rapid response mechanism that protects the proteome against elevated temperature and other stresses (Mittler et al., 2012). It is wildly accepted that a class of molecular chaperones, heat shock proteins (HSPs), are rapidly induced under the drive of heat shock transcription factors (HSFs) when plants are exposed to extremely high temperatures (Hu et al., 2009). In the HSR regulatory network, HSFs are the central components of the effective defense systems (Xue et al., 2014), whereas HSPs are directly responsible for protein folding, assembly, translocation, and degradation (Molinier et al., 2006).

Perennial woody plants are greatly significant components of the global ecosystem and play a key role in limiting the emissions of carbon dioxide (CO_2_) and other greenhouse gases. In addition, woody plants are the main biomass resources for biofuels. Due to the perennial and long life cycle, woody plants experience more severe and extreme abiotic stresses during their lives compared to herbaceous plants. Woody plants may have evolved more complex stress-responsive mechanisms (Anderegg et al., 2012). The ubiquitous and conserved HSR has been extensively studied in herbaceous plants. However, similar research studies on forest trees remain limited (Mittler et al., 2012). In this study, we review the recent progress on the roles of HSFs and HSPs and their regulatory network in the HSR of woody plants. Unraveling this underlying interconnected mechanism will help to understand the complex regulatory networks of the fitness and adaptive advantage of higher plants.

## Heat Shock Proteins: Master Players of HSR in Woody Species

Heat shock proteins are found in all living organisms and are classified into at least six different types based on their molecular weight: sHSPs (small heat stress proteins), HSP40s, HSP60s, HSP70s, HSP90s, and HSP100s (Zhang et al., 2013, 2015a; Zandalinas et al., 2018). Massive production of HSPs in plants is a major characteristic response for the acquisition of thermotolerance.

### Small Heat Stress Proteins

Small heat stress protein is a class of alpha-crystallin domain (ACD) chaperons with a molecular mass of 15–30 kDa. According to the sequence homology and subcellular localization, sHSPs are classified into 11 different classes. Class I–VI sHSPs are localized in the nucleus or cytoplasm, and the rest five classes of sHSPs are localized in mitochondria, chloroplasts, peroxisomes, or ER (Waters et al., 2008). The diversity of plant sHSPs reflects the molecular adaptability to various biotic and abiotic stresses (Hilton et al., 2012).

Until now, most studies have focused on the class I cytoplasmic sHSP and have divulged that sHSPs are involved in regulating thermotolerance in woody plants (Table 1), such as RcHSP17.8 in *Rosa chinensis* (*R. chinensis*) (Jiang et al., 2009, 2020), ThHSP18.3 in *Tamarix hispida* (*T. hispida*) (Gao et al., 2012), PtHSP17.8 in *Populus trichocarpa* (*P. trichocarpa*) (Li et al., 2016), CsHSP17.2 in *Camellia sinensis* (*C. sinensis*) (Wang et al., 2017a), and MsHSP16.9 in *Malus sieversii* (*M. sieversii*) (Yang et al., 2017). Among these genes, *RcHSP17.8* confers resistance to various stresses in *Escherichia coli* (*E. coli*), yeast, and *Arabidopsis* (Jiang et al., 2009). In addition, overexpression of *RcHSP17.8* in transgenic tobacco seedlings exhibits significant resistance to high temperatures and osmotic stresses, manifested by low electrolyte leakage and higher proline content under stress conditions (Jiang et al., 2020). Heterologous expression of *ThHSP18.3* protects yeast cells from salt, drought, heavy metals, and extreme temperatures (Gao et al., 2012). Overexpression of *PtHSP17.8* in *Arabidopsis* increases survival rate and root length under heat and salt stresses (Li et al., 2016). The *CsHSP17.2* in *C. sinensis* acts as a molecular chaperone to mediate heat tolerance by maintaining maximum photochemical efficiency and protein synthesis, enhancing the scavenging of reactive oxygen species (ROS), and inducing the expression of heat-responsive (HR) genes (Wang et al., 2017a). Overexpression of *M. sieversii MsHSP16.9* in *Arabidopsis* improves the tolerance of a plant to heat by alleviating the damages of ROS and regulating the expression levels of stress-related genes (Yang et al., 2017). Moreover, cytoplasmic class II and III sHSPs have also been reported to be involved in the HSR in woody species. For example, *SpHSP17.3*, a cytoplasmic class II *sHSP* gene from *Sorbus pohuashanensis* (*S. pohuashanensis*), responds to high temperature, salt, and drought stresses and has a certain effect on the adaptability of introduction and domestication (Zhang et al., 2020). Overexpression of a *Prunus mume* (*P. mume*) cytoplasmic class III sHSP gene (*PmHSP17.9*) improves the thermotolerance of transgenic *Arabidopsis* by enhancing superoxide dismutase (SOD) activity (Wan et al., 2016). Transient overexpression of a *Juglans regia* (*J. regia*) sHSP gene *JrsHSP17.3* in leaves enhances tolerance to cold, heat, and salt stresses by scavenging the accumulation of ROS and by accumulating osmotic adjustment substances (Zhai et al., 2016). In addition, the expressions of chloroplast small HSPs *CsHSP17.7, CsHSP18.1*, and *CsHSP21.8* (cytoplasmic classes I, II, and IV, respectively) from *C. sinensis* could be highly induced by heat and cold stresses. Overexpression of these small *CsHSPs* confers heat and cold tolerances in transgenic *Pichia pastoris (P. pastoris)* and *Arabidopsis* (Wang et al., 2017b). These studies indicate that sHSPs exhibit considerable influence in the adaptation of woody plants under heat and other abiotic stresses, which may be related to the long-term adaptive evolution of woody species.

**Table 1:** Summary of the *HSP* and *HSF* genes involved in heat stress in woody plants.

**Gene family**	**Gene symbol**	**Identified from species**	**Studied in species**	**Description**	**References**
sHSP	*RcHSP17.8*	*Rosa chinensis*	*Escherichia coli*, *Yeast,* *Arabidopsis thaliana*, *Nicotiana tabacum*	Induced by heat and osmotic stresses; positive regulator of high temperature, drought, salt, and mannitol stresses	Jiang et al., 2009, 2020
	*ThHSP18.3*	*Tamarix hispida*	*Yeast*	Induced by heat and cold stresses; positive regulator of salt, drought, heavy metals, cold, and heat stresses	Gao et al., 2012
	*PmHSP17.9*	*Prunus mume*	*Arabidopsis thaliana*	Induced by ABA, heat, salt, drought, and oxidative stresses; positive regulator of heat stress	Wan et al., 2016
	*PtHSP17.8*	*Populus trichocarpa*	*Arabidopsis thaliana*	Induced by ABA, heat, cold, salt, PEG, and oxidative stresses; positive regulator of heat and salt stresses	Li et al., 2016
	*JrsHSP17.3*	*Juglans regia*	*Yeast*, *Juglans regia*	Induced by heat, cold, and salt stresses; positive regulator of salt, cold, and heat stresses	Zhai et al., 2016
	*CsHSP17.2*	*Camellia sinensis*	*Escherichia coli*, *Pichia pastoris*, *Arabidopsis thaliana*	Induced by heat, PEG, and salt stresses; positive regulator of heat stress	Wang et al., 2017a
	*MsHSP16.9*	*Malus sieversii*	*Arabidopsis thaliana*	Induced by heat stress; positive regulator of heat stress	Yang et al., 2017
	*CsHSP17.7* *CsHSP18.1* *CsHSP21.8*	*Camellia sinensis*	*Yeast*, *Arabidopsis thaliana*	Induced by heat and cold stresses; positive regulator of cold and heat stresses	Wang et al., 2017b
	*SpHSP17.3*	*Sorbus pohuashanensis*	*Arabidopsis thaliana*	Induced by heat, salt, and drought stresses; positive regulator of salt stress	Zhang et al., 2020
HSF	*VpHSF1*	*Vitis pseudoreticulata*	*Nicotiana tabacum*	Induced by heat, drought, and pathogen *Erysiphe necator*; negative regulator of basal thermotolerance, osmotic stress, and pathogen; positive regulator of acquired thermotolerance	Peng et al., 2013

In contrast, the organelle-localized sHSPs have been more studied in herbaceous plants. For example, the mitochondria-localized *GhHSP24.7* in cotton positively regulates seed germination through modulating the generation of ROS in a temperature-dependent manner (Ma et al., 2019). The chloroplast-localized *AsHSP26.8a* in creeping bentgrass plays a negative role in abiotic stresses through both abscisic acid (ABA)-dependent and ABA-independent signaling pathways and other stress signaling pathways (Sun et al., 2020). In *Arabidopsis*, an endoplasmic reticulum (ER)-localized sHSP, *sHSP22*, is involved in ABA and auxin signaling crosstalk (Li et al., 2018). However, the functional study of the organelle-localized sHSP in woody species has not been reported. In the future, more attention should be paid to these genes to reveal the response mechanisms of different organelle levels to high temperatures and other abiotic stresses in woody plants.

### Other HSPs

Compared with the *sHSP* genes, members in other *HSP* gene families, such as *HSP40, HSP60, HSP70, HSP90*, and *HSP100*, have not been well-studied in woody species. There is a lack of results about individual gene function validated through genetic modification, although the genome-wide identification of such genes has been performed in several woody species. For example, Zhang et al. (2013, 2015a) have provided a comprehensive analysis of the gene organization and expression of *Populus HSF* and other *HSP* genes, such as *sHSP, HSP60, HSP70, HSP90*, and *HSP100*, under different abiotic stresses. A complex transcriptional regulatory network between *Populus HSFs* and *HSPs* has been generated based on their transcription patterns in poplar.

Several studies indicated that members in these HSP families play pivotal roles in regulating the thermotolerance in herbaceous species. For instance, knockdown of *CaHSP60-6* through virus-induced gene silencing in pepper [*Capsicum annuum* L. (*C. annuum* L.)] increases the heat sensitivity, which is manifested in the higher accumulation of ROS and lower membrane stability in silenced plants (Haq et al., 2019). Ectopic expression of a chrysanthemum *CgHSP70* in *Arabidopsis* enhances tolerance to heat, drought, or salinity, thereby protecting the plants from total damage (Song et al., 2014). Cytosolic *CaHSP70-1* from *C. annuum* is involved in the HSR through signal transduction pathways including calcium (Ca^2+^), hydrogen peroxide (H_2_O_2_), and putrescine (Guo et al., 2014). In addition, overexpression of a soybean *GmHSP90A2* improves thermotolerance in *Arabidopsis* (Huang et al., 2019). However, the functional analysis of these high molecular weight HSPs in woody species is rarely reported. In order to explore the adaptability of woody species to elevated temperatures, more in-depth studies are needed.

## Heat Shock Transcription Factors are the Main Regulators of HSPs

The expression of the *HSP* genes is mainly regulated by HSFs. HSF/HSP transcriptional module-based HSR was recognized as an evolutionally conserved mechanism coping with heat stress (Scharf et al., 2012). Plants have evolved more complicated HSR compared to yeast and animals, for example, the HSF family of plants comprised of 18–52 members while yeast and *Drosophila* only have a single copy, and mammals have four HSFs (Andrási et al., 2020). Despite significant variability in size and sequence of HSFs, their structures and functions are highly conserved across plant species (Lin et al., 2011). HSFs have a modular structure comprising a DNA-binding domain, an oligomerization domain, and a C-terminal activation domain (Scharf et al., 2012). Based on the variations in these three domains, especially the oligomerization domain, HSFs of plants can be divided into three classes (A, B, and C). Because of the vital regulatory responses to different stresses and developmental processes, the *HSF* gene family has been extensively characterized in the model plant *Arabidopsis*, as well as in several woody plants such as apple (*M. domestica*) (Giorno et al., 2012), poplar (*P. trichocarpa*) (Zhang et al., 2015a; Liu et al., 2019), desert poplar (*P. euphratica*) (Zhang et al., 2016), willow [*Salix suchowensis* (*S. suchowensis*)] (Zhang et al., 2015b), pear [*Pyrus bretschneideri* (*P. bretschneideri*)] (Qiao et al., 2015), tea (*C. sinensis*) (Liu et al., 2016), and grape [*Vitis vinifera* (*V. vinifera*)] (Liu et al., 2018). In *Arabidopsis*, rice, and tomato, *HSFA2* is strongly induced when plants are exposed to long-term heat stress or repeat-cycled heat stress and recovery (Scharf et al., 1998; Charng et al., 2007; Nishizawa-Yokoi et al., 2009), whereas *HSFA1a* seems to play a unique function as a master regulator of acquired thermotolerance and cannot be replaced by any other HSFs in tomato (Mishra et al., 2002). This implies that despite the existence of a certain conservative mechanism, HSF in different species may have functional specialization. Therefore, it is necessary to further study the gene function of HSF in various woody plants.

Transient overexpression of *Betula platyphylla* (*B. platyphylla*) *BpHSFA4* gene in leaves improves the salt stress tolerance by increasing the ability to scavenge ROS, thereby reducing cell damage or cell death and enhancing birch resistance (Liu et al., 2020). In contrast to the activity of the transcriptional activation of class A HSFs, the class B HSF proteins lack the activation domain and have a repression domain in the C-terminus. Peng et al. (2013) identified a novel class B2 *HSF* gene from Chinese wild *Vitis pseudoreticulata, VpHSF1*, which plays a key role in biotic and abiotic stress responses. Overexpressing *VpHSF1* in tobacco reduces the basal thermotolerance, improves the acquired thermotolerance, and enhances its susceptibility to osmotic stress and pathogen *Phytophthora parasitica* var. *nicotianae* Tucker (Table 1). Compared with herbaceous plants, the functional studies of HSFs in woody plants are still limited. *CpHSFB1* from *Carica papaya* (*C. papaya*) (Tarora et al., 2010) and *PsHSFB1* from *Paeonia suffruticosa* (*P. suffruticosa*) (Zhang et al., 2014) have been cloned from woody species, but their detailed biological functions have not been reported. Further unraveling the biological functions of each *HSF* can provide a better understanding of how woody plants can better cope with stresses during evolution.

## HSFs and HSPs Form a Complex Regulatory Network in HSR

**Figure 1:**
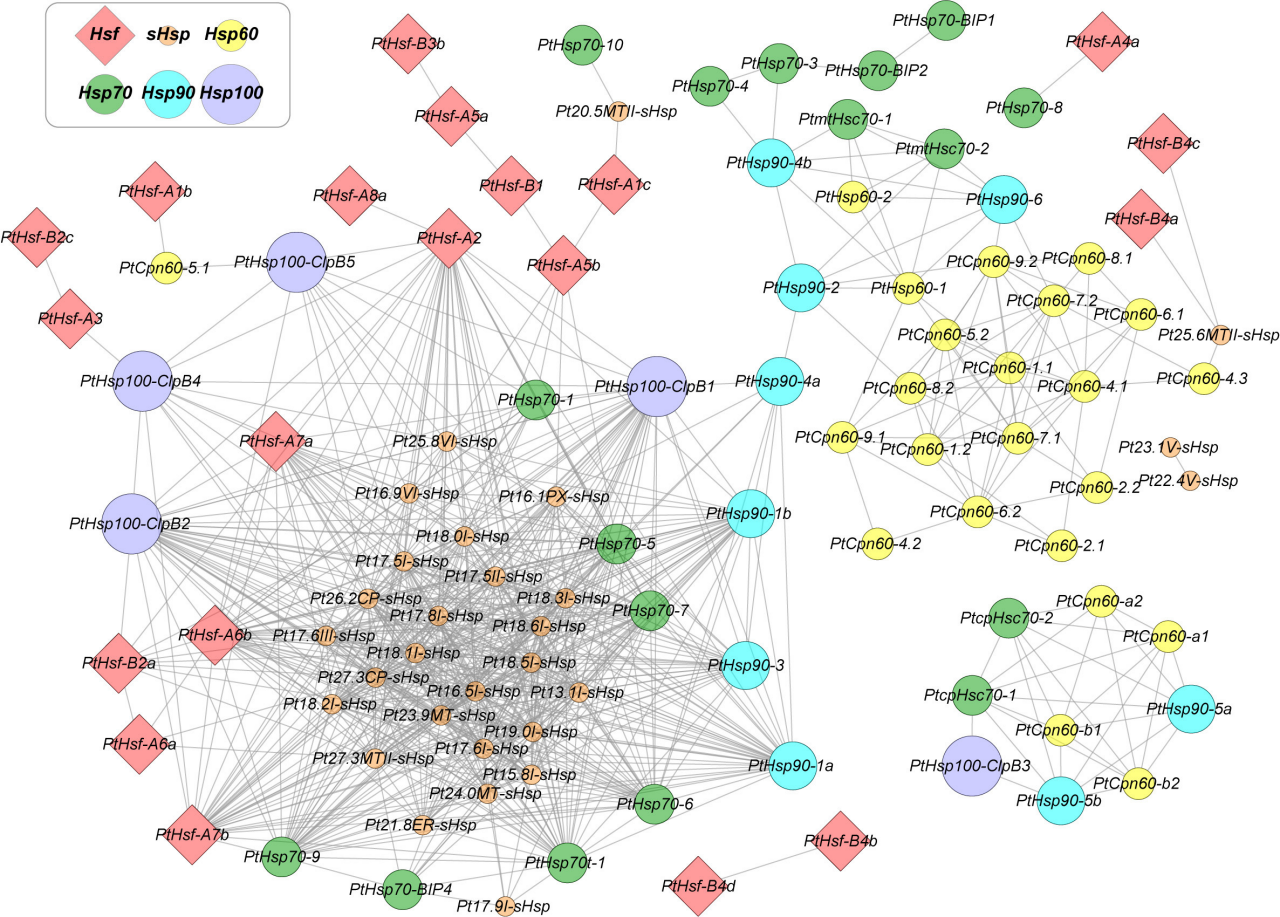
Co-expression network of the *HSF* and *HSP* genes in *Populus*. Nodes represent *HSFs* and *HSPs* genes in *Populus*, and edges indicate pairwise correlation constructed by weighted gene co-expression network analysis (WGCNA). Node color codes represent different gene families. Red diamonds indicate HSFs, and solid circles with orange, yellow, green, cyan, and purple indicate *sHSPs, HSP60s, HSP70s, HSP90s*, and *HSP100s*, respectively. Lines represent the co-expression interaction. The network was created using Cytoscape.

Plants possess a complex HSR regulatory network consisting of multiple *HSP* and *HSF* genes. HSFs contain a conserved DNA-binding domain at the N-terminus, which can recognize the DNA motif, 5′-nGAAnnTTCn-3′. This heat shock element (HSE) motif is commonly found in the promoters of HSF target genes (Andrási et al., 2020). Hierarchical transcriptional network among HSFs, HSPs, and other heat stress-responsive genes has been well-built in the model plant *Arabidopsis*. HSFA1s are the master regulators that respond quickly to heat stress, and other transcription factors, such as HSFA2 and DREB2A, were directly activated by HSFA1s (Liu et al., 2011; Yoshida et al., 2011). HSPs and ROS-scavenging enzymes are the main heat stress-induced proteins regulated by HSFs. They are required for protein quality control and oxidative homeostasis under heat–stress conditions (Ohama et al., 2017). Compared to *Arabidopsis*, woody species have very limited studies on HSFs. Salt-inducible PeHSF from *P. euphratica* directly binds to the HSE motifs in the *PeWRKY1* promoter and regulates its expression (Shen et al., 2015). HSFs regulate the expression of HSPs, and in turn, HSPs can physically interact with HSF proteins to affect the function of HSFs. In *Arabidopsis*, HSP90 and immunophilin ROF1 form a complex that co-imports HSFA2 into the nucleus and enhances its transcriptional activator activity under heat stress (Aviezer-Hagai et al., 2007; Meiri and Breiman, 2009). Another heat-inducible immunophilin ROF2, which is also a target of HSFA2, is recruited into the nucleus during accumulation and heterodimerizes with ROF1 in the complex, resulting in the inhibition of HSFA2. Therefore, the activity of HSFA2 in *Arabidopsis* is regulated by the two co-chaperones in both positive and negative manner by interacting with HSP90 (Meiri et al., 2010). In tomatoes, the constitutively expressed HSFA1a remains inactive in the complex formed with HSP70 and HSP90 under normal conditions. In contrast, the interaction of HSP90 with HSFB1 usually keeps the steady-state level of HSFB1 at a low level and targets HSFB1 to the degradation pathway of the 26S proteasome (Hahn et al., 2011). In addition, *V. vinifera VvGOLS1* has been heterologously expressed in *E. coli*, showing that it encodes a functional galactinol synthase. Transient expression assays showed that the heat stress factor VvHSFA2 transactivates the promoter of *VvGOLS1* in a heat stress-dependent manner (Pillet et al., 2012). However, the relationship between HSF and HSP, especially in woody species, remains unknown. With the rapid development of transcriptome-sequencing technology and the increase of big data in public databases, it is possible to use gene co-expression networks to explore the potential regulatory or interaction relationships between proteins. In this study, we use poplar as an example to construct an HSF-HSP co-expression network, due to the easy access to tremendous public expression data. As shown in Figure 1, we identified strong co-expressive relationships between *HSP90* (*PtHSP90-1a* and *PtHSP90-1b*) and *HSFA2* (*PtHSF-A2*), which is consistent with their protein–protein interactions in *Arabidopsis* and tomato (Meiri et al., 2010). This implies that HSR in woody species may share similar mechanisms across plant species. However, it is still an open question whether specific regulatory mechanisms or interaction relationships between HSFs and HSPs exist in woody plants.

## Future Perspectives

With ongoing greenhouse gas emissions, the ambient temperature is expected to rise over time. Woody plants have evolved more complex stress response mechanisms to cope with changing environments than herbaceous plants to guarantee a longer lifespan (Anderegg et al., 2012). However, the current knowledge of the functions of *HSF* and *HSP* genes during HSR in woody plants is still lacking. On the one hand, the transcriptional regulation or interaction between HSF and HSP in woody plants is unclear. Zhang et al. (2018) used the joint analysis of expression quantitative trait loci (eQTL) and co-expression network based on the *P. trichocarpa* natural variant populations to successfully identify the upstream regulatory transcription factor *WRKY* that controls the expression of the *HCT2* gene. This strategy provides a new method to identify the transcriptional regulatory relationship between genes. Therefore, the public gene expression database of woody plants can be used to preliminarily examine whether there is a potential regulatory or interaction relationship between HSFs and HSPs in these species. In addition, the continuous maturity of ChIP-Seq, CUT&Tag-Seq, and other technologies have effectively improved the ability to analyze the interaction between protein and DNA. The recently developed reverse-ChIP technology (Wen et al., 2020) can reversely mine the upstream regulators of specific target genes. In the future, the combined use of these methods can provide technical supports for the clarification of gene regulatory relationships in the HSR of woody plants. On the other hand, the traditional genetic transformation system is unstable and time-consuming for many woody species, which leads to insufficient research studies on the function of individual genes. Most of the studies just rely on the ectopic expression of *HSF* or *HSP* genes obtained from woody species in the model plant *Arabidopsis* or yeast. Therefore, fast and efficient gene function validation systems are urgently needed. The protoplast transient expression system (Zhang et al., 2018) can be used to quickly verify the relationship between HSFs and HSPs. And the recently developed genome editing technology has been successfully applied in many woody species (Li et al., 2021). However, it is necessary to further improve the efficiency and accuracy of gene editing to overcome the limitation of its application in woody species. In addition, future research studies should also pay attention to the signals and cascade transduction pathways of woody plants after sensing high temperatures, how these signals further activate the HSR, and how the posttranscriptional and posttranslational regulatory pathways participate in the HSR of woody species. The combined use of these methods can provide a basis for further in-depth analysis of the function of HSFs and HSPs in HSR in woody plants.

## Author Contributions

JZ conceived the study. JZ, FT, and X-LH drafted the manuscript. TY, XY, J-GC, and M-ZL revised the manuscript. All authors contributed to the article and approved the submitted version.

## Conflict of Interest

The authors declare that the research was conducted in the absence of any commercial or financial relationships that could be construed as a potential conflict of interest.
